# Age-related changes in function and gene expression of the male and female mouse bladder

**DOI:** 10.1038/s41598-018-20406-0

**Published:** 2018-02-01

**Authors:** Jun Kamei, Hiroki Ito, Naoki Aizawa, Harumi Hotta, Toshio Kojima, Yasunori Fujita, Masafumi Ito, Yukio Homma, Yasuhiko Igawa

**Affiliations:** 1 Department of Continence Medicine, The University of Tokyo Graduate School of Medicine, Tokyo, Japan; 2 Department of Urology, The University of Tokyo Graduate School of Medicine, Tokyo, Japan; 3 Department of Autonomic Neuroscience, Tokyo Metropolitan Institute of Gerontology, Tokyo, Japan; 4 Health Care Center, Toyohashi University of Technology, Aichi, Japan; 5 Research Team for Mechanism of Aging, Tokyo Metropolitan Institute of Gerontology, Tokyo, Japan; 6 Department of Urology, Japan Red Cross Medical Center, Tokyo, Japan

## Abstract

We investigated age-related changes in *in vivo* and *in vitro* functions and gene expression of the bladder of male and female mice. Mature and aged (12 and 27–30 month old) C57BL/6 mice of both sexes were used. Frequency volume, conscious free-moving cystometry and detrusor contractile and relaxant properties in *in vitro* organ bath were evaluated. mRNA expression level of muscarinic, purinergic, and β-adrenergic receptors and gene expression changes by cDNA microarray analysis of the bladder were determined. Cystometry demonstrated storage and voiding dysfunctions with ageing in both sexes. Detrusor strips from aged mice showed weaker contractile responses particularly in the cholinergic component and weaker relaxant responses to isoproterenol. These age-related impairments were generally severer in males. mRNA expression of bladder tissue was decreased for M3 muscarinic receptors in aged males and β2-adrenoceptors in aged females. cDNA microarray analysis results, albeit substantial sex difference, indicated “cell-to-cell signaling and interaction” as the most common feature of age-related gene expression. In summary, aged mice demonstrated voiding and storage dysfunctions resembling to detrusor hyperactivity with impaired contractility (DHIC), which were more pronounced in males. Genomic changes associated with aging may contribute to the age-related bladder functional deterioration in mice.

## Introduction

The rapid aging of population led by declining birth rate and mortality is a major global demographic trend^1^. Accordingly, the prevalence of aging-related diseases has been increasing^2^. In the urological field, lower urinary tract symptom (LUTS) and associated urodynamic changes, detrusor hyperactivity with impaired contractility (DHIC) in particular, is a common feature observed among the elderly^3–5^. However, these age-related changes have significant inter-individual variability and sex-related disparity^3,6^. The age-related changes or differences should be derived from genetic or epigenetic pathophysiology, which has not been fully investigated^3,7–9^. Previous human studies revealed decreased mRNA expression of M3 muscarinic receptor and impaired cholinergic neurotransmission with compensatory purinergic transmission in the detrusor smooth muscle of aged subjects^10,11^.

Several animal studies using rodents so far have explored the age-related changes in the bladder function^12–16^; *in vitro* intrinsic contractile property and contractile responses to cholinergic stimulation of detrusor smooth muscle were impaired^13,16^; cystometry in aged rats showed increased residual volume despite maintained maximum pressure^12,14^. However, most animal studies used only males or females, with a limited or different set of experiments (for example with or without anesthesia, etc.). Consequently, age-related changes in the bladder function showed inconsistent results even among the studies using the same type of animals, and are unknown for sex-based difference.

In the present study, we investigated age-related changes and differences of bladder functions *in vivo* and *in vitro* and gene expression in the male and female mouse bladder.

## Results

### Body weight, bladder weight, blood glucose, and sex hormones

Body weight in aged mice (AGED: 27–30-month old) was significantly lower compared to mature mice (MAT: 12-month old) in males alone. Bladder weight was counted for all mice excepting the mice used for cystometry (CMG) measurements (MAT: n = 13 and AGED: n = 9 in males, MAT: n = 13 and AGED: n = 14 in females). Only in males, both the actual and adjusted (by body weight) bladder weights of AGED mice, were significantly higher than those of MAT mice. Blood glucose level was not different between the two groups in either sex (Table 1A). Serum sex hormone data were obtained from 5 MAT and 4 AGED males and 5 MAT and 7 AGED females. Testosterone level was undetectable (less than 0.03 ng/ml) in 3 out of 4 AGED male mice and markedly reduced in the remaining one mouse (0.05 ng/ml) compared with MAT male mice (1.63 ± 0.92 (0.11–3.96) ng/ml). In contrast, estradiol level in female mice was not significantly different between MAT and AGED mice (Table 1).

**Table 1:** Body and bladder weights and blood glucose and sex hormone levels and FV measurement parameters in mature (MAT) and aged (AGED) mice.

**Body and bladder weight and blood examinations**	**Male**		p value	**Female**		p value
	MAT	AGED		MAT	AGED	
Body weight (g)	33.27 ± 0.63 (n = 20)	29.80 ± 0.58(n = 16)	<0.001^***^	27.77 ± 0.66 (n = 19)	26.66 ± 0.40 (n = 20)	0.16
Bladder weight (g)	38.92 ± 1.37 (n = 13)	54.0 ± 3.94 (n = 9)	0.0048^**^	33.3 ± 1.08 (n = 13)	36.93 ± 1.80 (n = 14)	0.10
Bladder weight/body weight	1.16 ± 0.047 (n = 13)	1.77 ± 0.13 (n = 9)	0.0010^**^	1.24 ± 0.056 (n = 13)	1.39 ± 0.073 (n = 14)	0.11
Blood glucose level (mg/dL)	155.2 ± 10.9 (n = 13)	166.1 ± 55.3 (n = 9)	0.82	125.8 ± 6.8 (n = 13)	115.7 ± 9.0 (n = 14)	0.38
Serum testosterone level (ng/mL)	1.63 ± 0.92 (n = 5)	0.05 (n = 1) ^#^	N/A	Not evaluated	Not evaluated	
Serum estradiol level (pg/mL)	Not evaluated	Not evaluated		27.0 ± 4.1 (n = 5)	20.3 ± 3.9 (n = 7)	0.28
**FV measurement parameters**	MAT (n = 10)	AGED (n = 8)	p value	MAT (n = 10)	AGED (n = 7)	p value
Voiding frequency (times/day)	6.10 ± 1.14	34.5 ± 6.16	0.002^**^	9.9 ± 1.68	10.0 ± 2.17	0.97
Total voided volume (ml/day)	1.56 ± 0.15	6.85 ± 0.86	0.003^**^	1.81 ± 0.24	2.57 ± 0.18	0.03^*^
Mean voided volume (μl)	332.7 ± 52.8	225.9 ± 32.9	0.12	194.8 ± 16.9	325.6 ± 66.2	0.099
Mean flow rate (μl/sec)	61.7 ± 3.1	54.0 ± 3.0	0.09	68.0 ± 8.7	46.6 ± 6.0	0.003^**^
Water intake (ml/day)	4.12 ± 0.34	11.86 ± 1.06	<0.001^***^	4.45 ± 0.65	5.83 ± 0.65	0.17
Food intake (g/day)	4.38 ± 0.60	5.25 ± 0.47	0.31	4.09 ± 0.85	5.38 ± 0.38	0.25

*p < 0.05, **p < 0.01, ***p < 0.001: Significantly different from MAT mice of the same sex (unpaired t-test)

^#^Serum testosterone levels in three AGED male mice were undetectable (<0.03 ng/ml).

N/A: not applicable.

### Frequency/volume (FV) measurements

Compared with MAT mice, AGED mice showed significantly increased total voided volume in both sexes, higher voiding frequency and increased water intake in males, and lower mean flow rate in females. There were no significant differences in voided volume per micturition and food intake between the two groups in either sex (7–10 mice per group) (Table 1).

### Conscious free-moving CMG measurements

**Figure 1:**
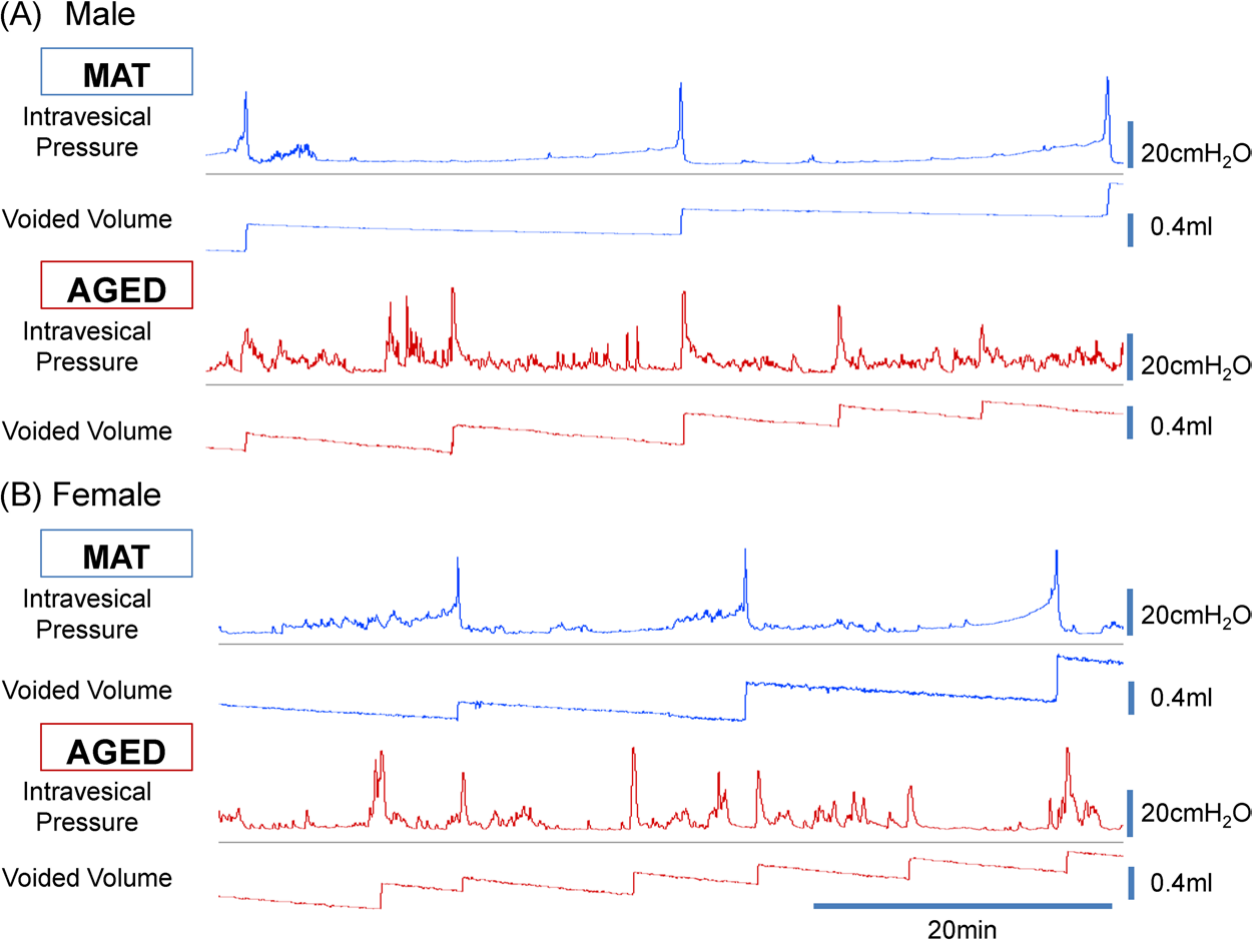
Representative traces of intravesical pressure and voided volume during conscious cystometry with saline instillation (15 µl/min) from each group in male (**A**) and female (**B**) mice. MAT: mature (12-month old), AGED: aged (27–30-month old).

We had allocated 7 males and 6 females of each group for CMG but evaluated 6 MAT and 5 AGED mice of both sexes. Two male AGED mice were dead before measurements. The CMG recordings of 1 male MAT mouse and 1 female AGED mouse were excluded, because reproducible tracings were not obtained. Compared with MAT mice, AGED mice showed multiple significant changes; (1) increased frequency of non-voiding contractions (NVCs) per minute in both sexes, (2) shorter intercontraction interval, smaller bladder capacity, and smaller voided volume in males, (3) lower mean flow rate in both sexes, and (4) lower maximum pressure in males (Table 2 and Fig. 1).

**Table 2:** Conscious free-moving CMG measurement parameters in mature (MAT) and aged (AGED) mice.

	Male			Female		
	MAT (n = 6)	AGED (n = 5)	p value	MAT (n = 6)	AGED (n = 5)	p value
Storage parameters						
Basal pressure (cmH_2_O)	3.83 ± 0.30	5.75 ± 1.03	0.14	4.39 ± 0.27	5.47 ± 0.46	0.07
Threshold pressure (cmH_2_O)	11.57 ± 1.07	9.29 ± 0.94	0.16	13.71 ± 2.31	9.11 ± 0.97	0.11
Intercontraction interval (sec)	1749.2 ± 98.7	847.2 ± 253.6	0.015*	1113.3 ± 103.1	759.2 ± 132.7	0.07
Voided volume (μl)	473.4 ± 25.0	247.3 ± 91.7	0.044*	290.1 ± 30.0	208.5 ± 40.4	0.12
Bladder capacity (μl)	480.3 ± 30.1	258.8 ± 87.4	0.046*	303.2 ± 33.1	225.87 ± 34.1	0.14
Bladder compliance (ml/cmH_2_O)	0.065 ± 0.010	0.077 ± 0.008	0.82	0.052 ± 0.019	0.125 ± 0.046	0.15
Number of NVCs (/min)	0.04 ± 0.02	0.43 ± 0.12	0.028*	0.03 ± 0.02	0.34 ± 0.10	0.037*
Amplitude of NVCs (cmH_2_O)	8.51 ± 2.03 (n = 3)	7.31 ± 0.64	0.51	8.08 ± 1.06 (n = 3)	9.32 ± 0.44	0.25
Voiding parameters						
Maximum pressure (cmH_2_O)	35.09 ± 1.77	28.18 ± 1.48	0.017*	31.72 ± 1.66	32.09 ± 2.06	0.89
Mean flow rate (μl/sec)	70.9 ± 9.0	43.8 ± 3.6	0.015*	73.8 ± 4.5	52.2 ± 3.4	0.005**
Residual volume (μl)	6.9 ± 1.3	11.5 ± 8.6	0.63	12.3 ± 7.3	17.3 ± 16.5	0.77
Voiding efficiency (%)	98.8 ± 0.3	91.8 ± 6.2	0.36	96.2 ± 1.8	91.4 ± 8.1	0.59

NVCs: non-voiding contractions

*p < 0.05, **p < 0.01: Significantly different from MAT mice of the same sex (unpaired t-test).

### *In vitro* functional analysis of detrusor strips

#### Contractile responses

**Figure 2:**
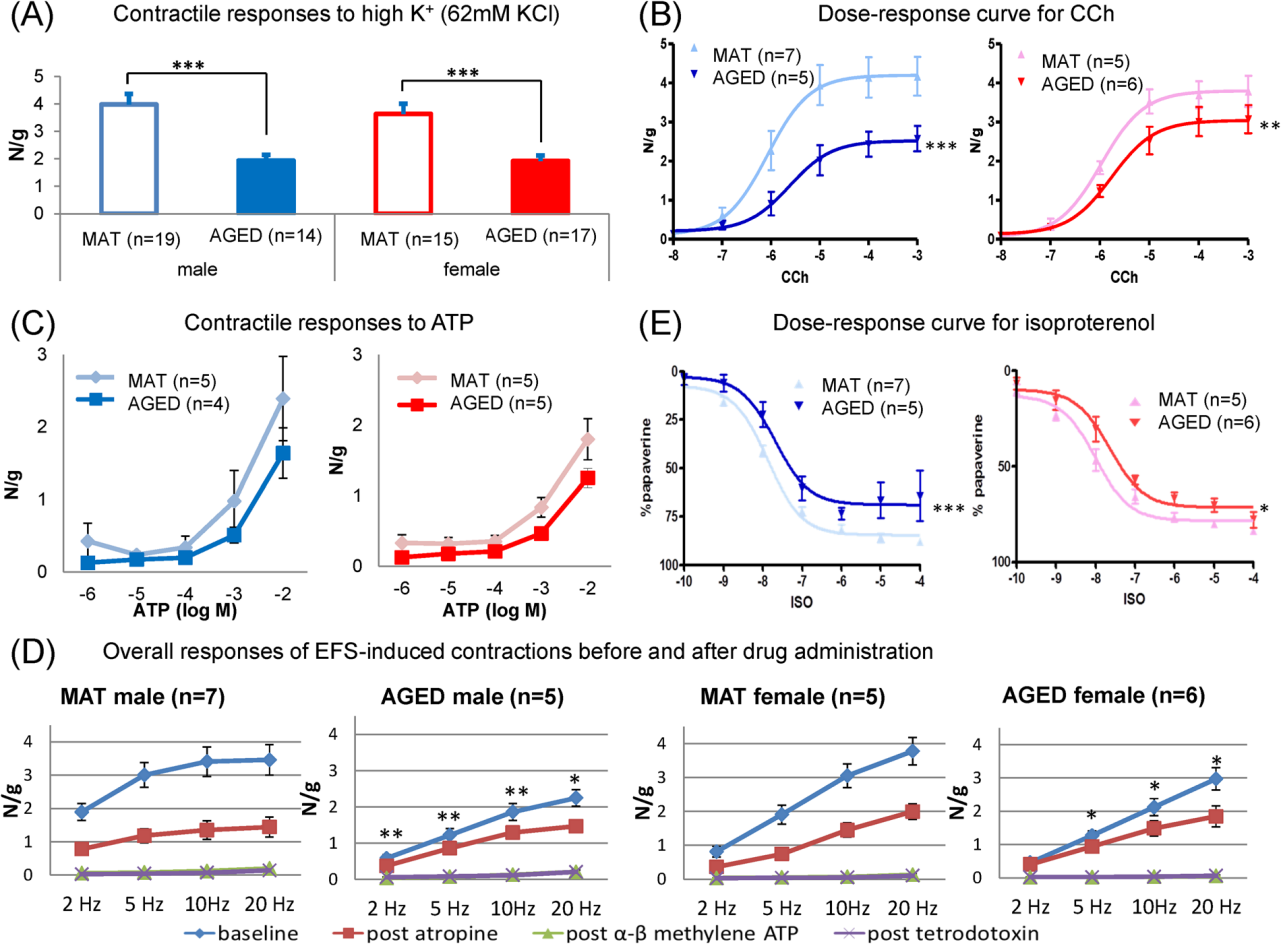
Contractile and relaxant responses of detrusor strips. Contractile responses to high K^+^ (**A**), CCh (**B**), ATP (**C**), and EFS (**D**) and relaxant responses to isoproterenol (**E**). MAT: mature (12-month old), AGED: aged (27–30-month old). Values are expressed as mean ± SEM. *p < 0.05, **p < 0.01, ***p < 0.001, significantly different from MAT of the same sex (**A**,**C**,**D**) unpaired t-test, (**B**,E**)** f-test of nonlinear regression).

In both sexes, the contractile responses to high K^+^ was weaker in AGED mice compared with MAT mice (assayed in 14 to 19 detrusor strips and 9 to 11 mice per group) (Fig. 2A). The Emax of the contractile responses to carbachol (CCh) in AGED mice was also significantly lower in both sexes (5 to 7 detrusor strips and 5 to 7 mice per group) (Fig. 2B). In contrast, the pEC50 values for the responses to CCh (male: 6.07 vs. 5.62, female: 5.97 vs. 5.76) and the responses to adenosine triphosphate (ATP) showed no significant age-related differences in either sex (4 or 5 detrusor strips and 4 or 5 mice per group) (Fig. 2B and C). The amplitude of contractions induced by electric field stimulation (EFS) was significantly lower in AGED mice in both sexes, except at 2 Hz in females (5 to 7 detrusor strips and 5 to 7 mice per group). However, after atropine administration, there were no significant differences between the two groups in either sex (Fig. 2D).

#### Relaxant responses

Compared with MAT mice, the maximum relaxant response to isoproterenol (ISO) was significantly weaker in AGED mice in both sexes, although its pEC50 value (male: 7.86 vs. 7.64, female: 7.94 vs. 7.61) was not significantly different between groups in either sex (Fig. 2E).

### Histological examinations

**Figure 3:**
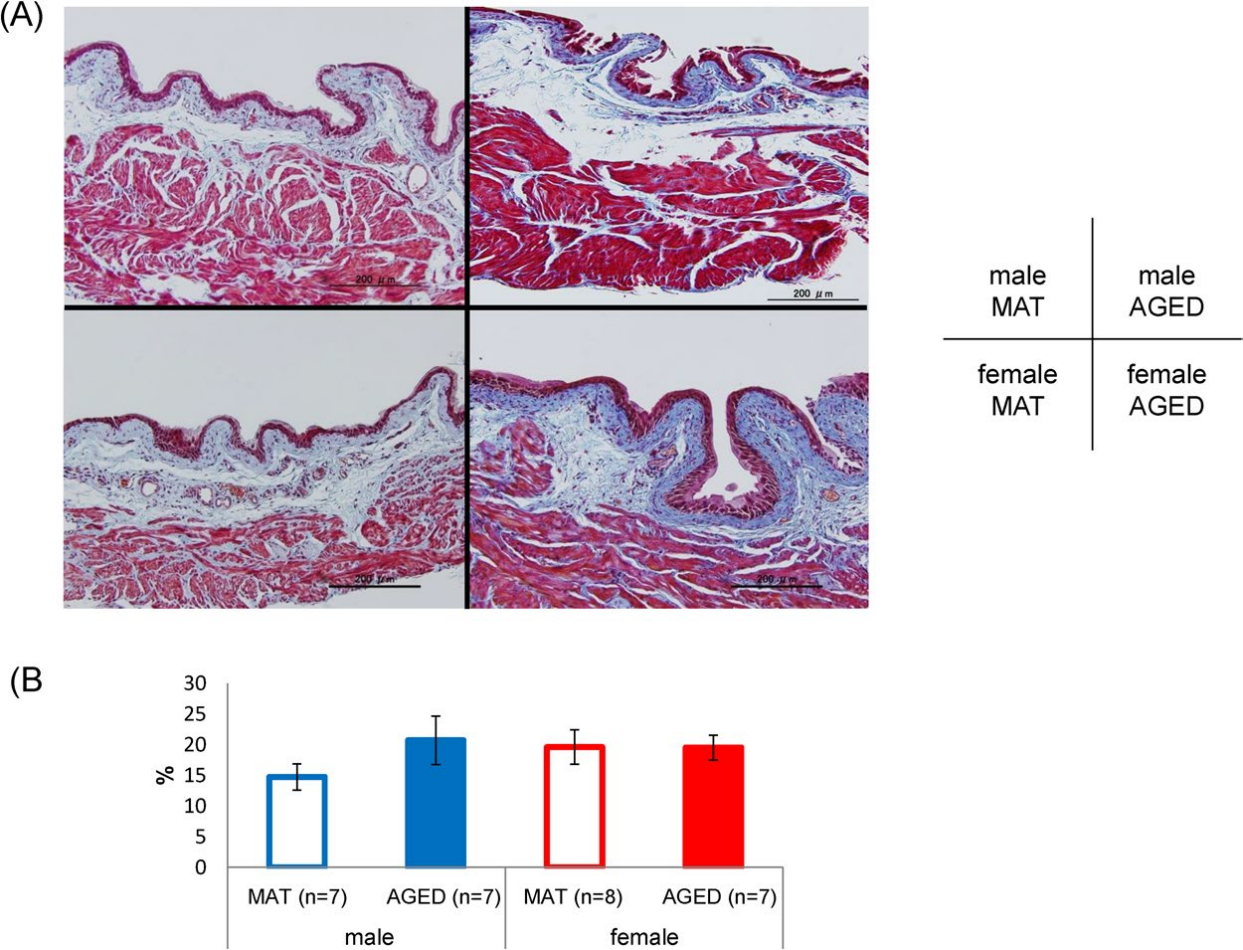
Representative histological images of bladder specimens taken from each group of mice, with Masson’s trichrome staining. MAT: mature (12-month old), AGED: aged (27–30-month old) Scale bar = 200 µm (**A**). Collagen deposition rate in the detrusor layer (**B**). No significant differences between MAT and AGED were found in either sex (unpaired t-test).

In males, collagen deposition in the detrusor layer tended to increase in AGED mice, but there were no significant differences in either sex (7 or 8 bladder specimens per group, male: *p* = 0.077, Fig. 3).

### Real-time reverse transcription polymerase chain reaction (RT-PCR)

**Figure 4:**
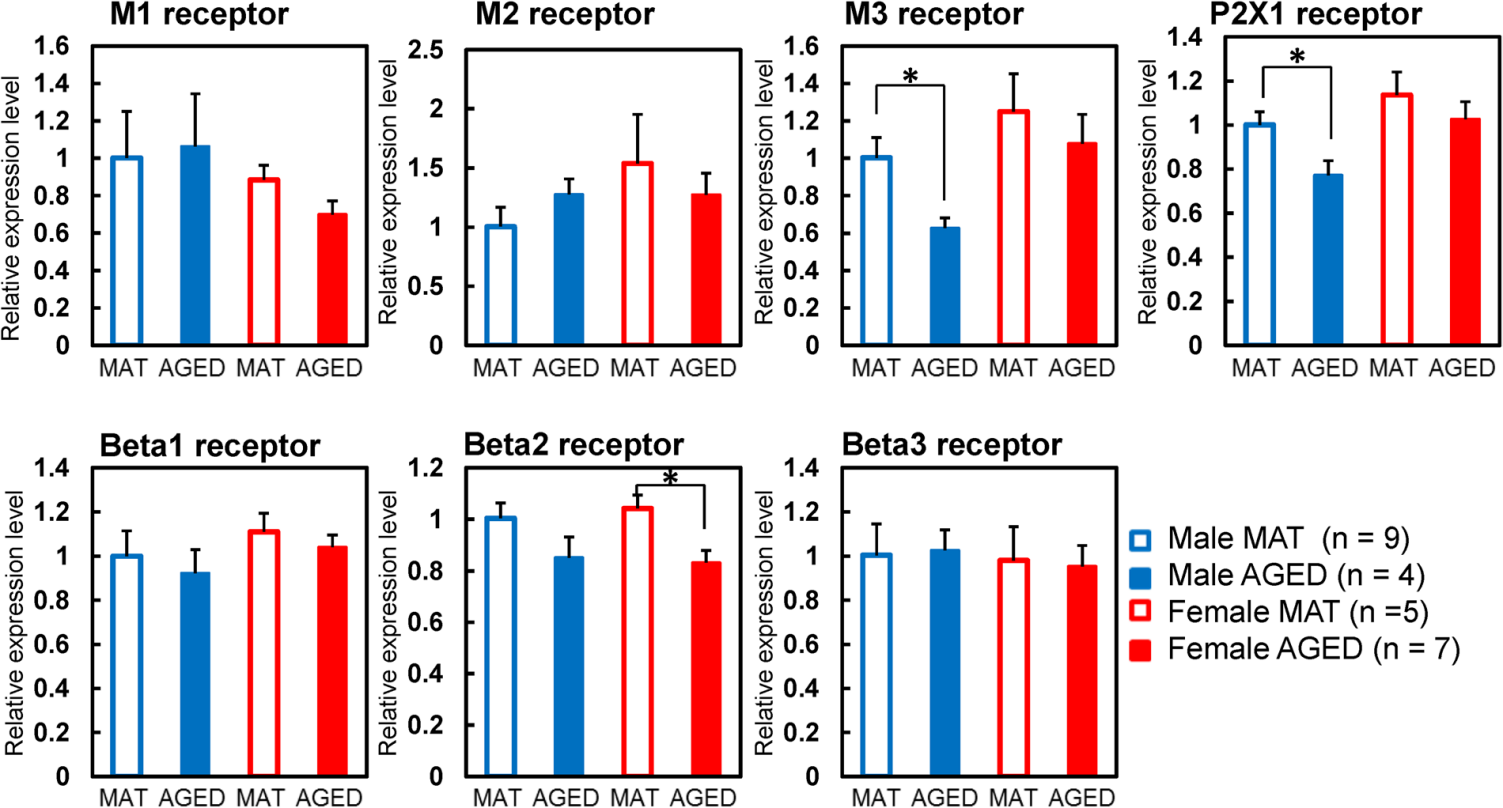
Receptor cDNA expression. MAT: matured (12-month old), AGED: aged (27–30-month old) *p < 0.05 significantly different from MAT of the same sex (unpaired t-test).

AGED bladder showed significantly lower M3 and P2X1 receptor expression in males and β2-adrenoceptor expression in females (MAT: n = 11 and AGED: n = 4 in males, MAT: n = 5 and AGED: n = 7 in females, Fig. 4). No significant differences were found in receptor expression of M1, M2, β1, or β3 in either sex (Fig. 4).

### cDNA microarray analyses

Total RNA from 4 bladders in each group were investigated. In AGED mice, 469 genes in males, 392 in females, and 8 in both sexes were significantly upregulated, whereas 544 genes in males, 75 in females, and 37 in both sexes were significantly downregulated. Of the top 5 upregulated genes (Tables 3), 4 genes were not identified as ontology terms, with Kcnj5 as an exception. In contrast, the top 5 downregulated genes were all associated with “response to stimuli” and “extracellular region” as per gene ontology analysis. When gene functions were classified into three categories (diseases and disorders, molecular and cellular functions, and physiological system development and function), the top 5 functions that significantly changed with aging in each category are summarized in Table 4. The genes that commonly changed with aging in both sexes were associated with “cell-to-cell signaling and interaction.”

**Table 3:** Top five genes of the urinary bladder upregulated and downregulated in aged mice compared with mature mice of the same sex.

	Gene Symbol	Entrez Gene Name	Fold change	
			Male	Female
Upregulated genes	AI593442	chromosome 11 open reading frame 87	10.07	3.36
	Kcnj5	potassium inwardly-rectifying channel, subfamily J, member 5	3.30	3.96
	Tmem200c	transmembrane protein 200 C	3.19	2.76
	D9Ertd115e	cytochrome P450, family 11, subfamily A, polypeptide 1	2.90	2.55
	Gsg1l	GSG1-like	2.77	2.19
Downregulated genes	Chil3	chitinase-like 3	45.45	6.32
	Defb50	defensin beta 50	9.29	3.55
	S100a9	S100 calcium binding protein A9	7.16	3.10
	F10	coagulation factor X	6.23	2.87
	Mmp3	matrix metallopeptidase 3	5.28	3.94

**Table 4:** Gene functions in the urinary bladder most significantly changed with aging.

Male	Female
Diseases and Disorders	
Cancer	Immunological Disease
Dermatological Diseases and Conditions	Antimicrobial Response
Organismal Injury and Abnormalities	Inflammatory Response
Gastrointestinal Disease	Endocrine System Disorders
Inflammatory Disease	Gastrointestinal Diseases
Molecular and Cellular Functions	
Lipid Metabolism	Cell Signaling
Small Molecule Biochemistry	Cell Morphology
Molecular Transport	Cellular Compromise
Drug Metabolism	Cellular Function and Maintenance
Cell-To-Cell Signaling and Interaction	Cell-To-Cell Signaling and Interaction
Physiological System Development and Function	
Behavior	Hematological System Development and Function
Nervous System Development and Function	Tissue Morphology
Endocrine System Development and Function	Immune Cell Trafficking
Organismal Development	Tissue Development
Organ Morphology	Skeletal and Muscular System Development and Function

## Discussion

The *in vivo* study results indicated that aged mice had storage and voiding dysfunctions, which would resemble to DHIC, and such dysfunctions were more pronounced in males. More specifically CMG showed an increased number of NVCs and lower mean flow rate in aged mice of both sexes, with related parameters more significantly impaired in males. These results were compatible with bladder dysfunctions commonly observed among the elderly^3,8,9^. Less obvious age-related changes in FV parameters would be explained by polyuria in aged mice, which complicated the evaluation of the bladder function by FV.

*In vitro* organ bath studies using isolated detrusor strips demonstrated decreased contractile responses to high K^+^ with aging in both sexes, suggesting impaired intrinsic contractile property of detrusor smooth muscle. The finding is consistent with previous reports using male mice and female guinea pigs^13,17^. Our contractile response studies also revealed lower atropine sensitive component in EFS-induced contractions and lower responses to muscarinic stimulation with aging in both sexes. In contrast, there is no significant change in purinergic component in EFS-induced contractions and contractile responses to purinergic stimulation. These results are in line with the previous findings demonstrating age-related impairment of contractile responses to both carbachol and EFS (muscarinic but not purinergic component) in the male rat and human bladders^10,14,16^. We have confirmed this age-related impairment in females as well. Thus, the age-related changes of bladder contractility would be attributable to impairment of cholinergic contractile properties of detrusor smooth muscle with aging in addition to impaired intrinsic contractile property. Our experiments also showed that the relaxant responses to ISO were attenuated in aged mice of both sexes, which was consistent with findings in the rat bladder^18,19^. We demonstrated, for the first time to our knowledge, using real-time RT-PCR that the expression of the M3 receptors in males and the β2-adrenoceptors in females was downregulated in aged mice. The results would support the functional changes demonstrated in the *in vitro* organ bath studies. Our results also suggest sex differences in age-related mRNA expression changes of muscarinic, purinergic, and β-adrenergic receptors of the bladder. In contrast, downregulated P2X1 receptor expression in aged male mice, which is consistent with observations in male human detrusor without bladder outlet obstruction^10,20^, was not reflected in *in vitro* contractile experiments. We did not fully address this discrepancy, which may be due to differences between genomic and functional contributions in the isolated bladder strips. Taken together, *in vivo* and *in vitro* studies, gene expression studies as well, indicate that both contractile and relaxant responses of detrusor smooth muscle were impaired with aging in both sexes. These observations in both *in vitro* and *in vivo* experiments suggest that our aged mice model would reproduce the aged human bladder condition.

The microarray analysis of the bladder revealed “cell-to-cell signaling and interaction” as the most common age-related changes in both sexes. Such genomic changes may be responsible for functional and morphological changes in the bladder such as collagen deposition, particularly in males. This should be addressed in more detail using our current findings as a basis for future studies. For example, determining the genes responsible for age-related bladder dysfunction would bring useful information for understanding and clinical management of age-related bladder dysfunction. Regarding sex disparity, male mice showed more severe aging-related functional deterioration than females, as per the CMG measurements, *in vitro* organ bath studies, and histological evaluations. A possible reason would be alteration in the sex hormone status with aging^21^. Serum testosterone levels were remarkably lower in AGED male mice compared with those in MAT male mice, whereas there was no significant difference in serum estradiol levels between AGED and MAT female mice. Keast *et al*. suggested that androgens are essential for the maturation and maintenance of the structure of pelvic autonomic neurons and pelvic ganglion neurons that supply the bladder of the male Wistar rat^22^.

Our study has some limitations. First, we investigated age-related changes only at two points of life. Further studies using other age groups can describe chronological changes of the bladder function more precisely, disclosing the time-dependent maturation and deterioration of the bladder. Second, we have not examined the changes of the properties of neuronal tissue like as dorsal root ganglia. Considering the relevance of neuronal control on bladder function, gene expression changes of the neuronal tissue may disclose more precisely age-related bladder dysfunction. Third, the mechanisms of age-related deterioration of bladder contractile or relaxant properties are to be disclosed. Further studies including evaluating the age-related differences of voltage gated calcium channels or potential intracellular pathways are needed. Forth, the implication of age-related genomic changes remained to be speculative. Studies using genetically engineered mice would contribute to elucidating the role of genes responsible for the functional changes observed. Finally, we have not examined pharmacological responses to therapeutic agents. The age-related changes in responsiveness, if any, should be examined further for improving the management of lower urinary tract dysfunctions according to sex or age.

In conclusion, aged mice demonstrated voiding and storage dysfunctions resembling to DHIC, which were more pronounced in males. Genomic changes associated with aging may contribute to the age-related functional deterioration in mice.

## Methods

### Animals and experimental groups

C57BL/6 male and female mice were divided into two groups: MAT (male: N = 20, female: N = 19) and AGED (male: N = 16, female: N = 20). Mice were maintained under standard laboratory conditions, with a 12:12 h light (9:00 a.m. to 9:00 p.m.) and dark (9:00 p.m. to 9:00 a.m.) cycle and free access to food pellets and tap water. Experimental protocols were approved by the Animal Ethics Committee of the University of Tokyo Graduate School of Medicine, Tokyo, Japan and were in line with NIH guidelines for the care and use of experimental animals. Mice were obtained from the Tokyo Metropolitan Institute of Gerontology, and after a 1-week adaptation period, they were used for measurements.

### FV measurements

Each mouse was separately placed, conscious and without restraint, in a MCM/TOA-UF001–006 metabolic cage (Mitsubishi Chemical Medience, Tokyo, Japan). This metabolic cage can pass urine and feces separately owing to a specially designed net, facilitating precise measurement of the voided urine volume^16,23^. After a 24-h adaptation, the voided volume, voiding frequency, duration of each micturition, and water intake volume were recorded with a PowerLab data acquisition system (AD Instruments, Sydney, Australia) continuously for 24 h starting from 9:00 am, with a 12:12 h light (9:00 a.m. to 9:00 p.m.) and dark (9:00 p.m. to 9:00 a.m.) cycle. Mean flow rate of each voiding was calculated by dividing voided volume by duration of micturition. The mice had free access to water and food during recordings.

### Conscious free-moving CMG measurements

In separate mice from other experiments, under isoflurane anesthesia, a polyethylene catheter (Clay-Adams PE–10, Parsippany, NJ) was implanted into the bladder through the dome and subcutaneously tunneled and exteriorized at the back of the neck. Five days after the surgery, conscious CMG measurements were performed without any restraint in the metabolic cage (MCM/TOA-UF001-006). The intravesical catheter was connected via a three-way stopcock to a pressure transducer (DX-100, Nihon Kohden, Tokyo, Japan) and a syringe pump (KDS 200, Muromachi Kikai Co. Ltd., Tokyo, Japan) for saline instillation at 15 µl/min. Intravesical pressure and voided volume were measured using PowerLab (AD Instruments). After stable and reproducible recordings were obtained, micturition cycles during 1 h were averaged and the following cystometric parameters were analyzed: basal pressure (minimum bladder pressure), threshold pressure (bladder pressure at the onset of micturition), maximum pressure (maximum bladder pressure during micturition), voided volume, intercontraction interval (duration between two micturitions), bladder capacity (instilled saline volume), residual volume (bladder capacity − voided volume), mean flow rate (voided volume divided by duration of micturition), voiding efficiency (voided volume divided by bladder capacity × 100%), bladder compliance [bladder capacity divided by (threshold pressure − basal pressure)], and number and amplitude of NVCs^24^. NVCs were defined as bladder contractions without micturition, with amplitudes of more than 5 cmH_2_O^25^. Mice using CMG measurements were euthanatized after measurements, and the bladder tissues of them were not used for other *in vitro* experiments, because catheter implantation may change the property of the bladder tissue.

### Blood glucose and sex hormone measurements

At approximately 1:00 pm, mice were restrained and blood glucose levels were measured in the tail vein using a disposable glucose test sensor (Glutest, Sanwa Kagaku Kenkyusho Co. Ltd, Tokyo, Japan). Mice were then anesthetized with an intraperitoneal injection of pentobarbital sodium (30 mg/kg). The peritoneal cavity was opened after central abdominal incision, and whole blood (approximately 0.4 ml) was harvested from the inferior vena cava. The blood sample was transferred to a test tube and left at room temperature for 30 min and at 4 °C for 16 h; subsequently, the sample was centrifuged at 1200 g for 25 min to separate the serum. The collected serum was stored frozen (−20 °C) until measurements. Serum testosterone levels for male by an electrochemiluminescence assay and estradiol levels for female mice by a chemiluminescence assay were analyzed by SRL, Inc. (Tokyo, Japan).

### In vitro studies for functional analysis using detrusor strips

This investigation was performed as described in our previous reports^16,26^. After mice were sacrificed by an overdose of pentobarbital sodium, the bladder bodies were harvested, and longitudinal full-thickness bladder strips were transferred to 5 ml organ baths. After a 2-h equilibration period with a stable tension of 10 mN for contractile and 5 mN for relaxant experiments, investigations were initiated. The strips were first exposed to a high K^+^ (62 mM KCl) Krebs solution. After 3 times washout, contractile responses to particular stimuli were examined in separate specimens, including 1: Cch (10^−8^ M to 10^−3^ M), 2: ATP (10^−6^ M to 10^−2^ M), and 3: EFS (pulse width: 0.8 ms, 50 V; pulse duration: 5 s; and stimulation interval: 1 m at 2, 5, 10, and 20 Hz). Cumulative application of Cch and ATP were given after a previous peak contraction without washout. After baseline measurements, EFS was repeated after 10^−6^ M atropine administration, purinoceptor desensitization by repeated 10^−5^ M α, β-Methylene-ATP (mATP) administrations, and finally, 10^−6^ M tetrodotoxin (TTX) administration. Each drug application interval was 2 minutes. In separate specimens, relaxant responses to ISO (10^−10^ M to 10^−4^ M) were evaluated under a mechanical 5 mN tension with 5 minutes interval. Papaverine (10^−3^ M) was applied 5 minutes after the maximal dose of ISO as a referent drug to induce relaxation. The contractile responses to CCh and EFS and the relaxant response to ISO were evaluated using detrusor strips in the same mice and the contractile responses to ATP were evaluated using the strips in separate mice. The amplitude was measured in all *in vitro* contractile and relaxant experiments.

### Histological examination

Isolated bladder body specimens were fixed in 4% paraformaldehyde-phosphate buffered saline, embedded in paraffin, and cut into 3-μm sections. Masson’s trichrome staining was performed to analyze fibrosis in the detrusor muscle layer. Collagen deposition was determined in 3 randomly selected sections^27^. Collagen deposition rate was defined as the collagen content (blue areas) divided by the muscle area × 100% in each section^16^. Images were analyzed using Adobe software and Image J (http://rsb.info.nih.gov/ij/)^16,28^.

### Real-time RT-PCR

The bladder body dissected out were immediately placed in RNA*later* RNA Stabilization Reagent (QIAGEN, Venlo, The Netherlands). Total RNA extracted from tissues using the miRNeasy Mini Kit (QIAGEN) was reverse transcribed to cDNA using the ReverTra Ace qPCR RT Master Mix (TOYOBO, Osaka, Japan) according to the manufacturer’s procedure. For relative quantification of mRNA expression, real-time PCR was performed using the THUNDERBIRD SYBR qPCR Mix (TOYOBO) and gene-specific primers on the StepOnePlus Real-Time PCR System (Thermo Fisher Scientific, Waltham, MA, USA). The data was normalized to Gapdh. Primer sequences were as shown in Supplementary table.

### cDNA microarray analyses

Total RNA from the bladder (200 ng) extracted for real-time RT-PCR was amplified and labeled with Cy3 using a Low Input Quick Amp Labeling Kit (Agilent Technologies, Santa Clara, CA, USA) according to the manufacturer’s instructions. The labeled cRNA was hybridized to the Agilent SurePrint G3 Mouse GE 8 × 60 K Microarray according to the manufacturer’s procedure. After hybridization, the microarrays were scanned on a DNA Microarray Scanner with Scan Control software. Raw data were collected from processed images using Feature Extraction software (Agilent). Gene expression analysis was performed with the Subio platform (Subio, Kagoshima, Japan). These data were compared using t-tests between MAT and AGED groups, with p < 0.05 considered statistically significant. The remaining probes were selected using the criterion of at least a 2-fold change. The gene list was analyzed using Ingenuity pathway analysis software (QIAGEN). The microarray data discussed in this publication have been deposited in NCBI’s Gene Expression Omnibus (GEO) and are accessible through the GEO Series accession number GSE100219 (https://www.ncbi.nlm.nih.gov/geo/query/acc.cgi?acc=GSE100219).

### Drugs

CCh, ATP, atropine, and TTX were purchased from Wako Chemical, Tokyo, Japan. ISO and mATP were purchased from Sigma-Aldrich (St. Louis, MO, USA). Papaverine was purchased from Cayman Chemical, Ann Arbor, MI, USA. Krebs solution comprised 118 mM NaCl, 4.7 mM KCl, 2.5 mM CaCl_2_, 25.0 mM NaHCO_3_, 1.2 mM KH_2_PO_4_, and 11 mM glucose (pH 7.4).

### Statistical Analysis

All data are expressed as the mean ± SEM. pEC50 and Emax of the CCh- and ISO- responses were analyzed by f-test of nonlinear regression, and other results were analyzed using unpaired t-test between MAT and AGED groups of the same sex. Statistical significance was considered at *p* values < 0.05.

## Electronic supplementary material

Supplementary table
