# Supplementary material for: Age-related changes in function and gene expression of the male and female mouse bladder
Source: Sci Rep. 2018 Feb 1;8:2089. doi: 10.1038/s41598-018-20406-0 (PMC5794976; doi:10.1038/s41598-018-20406-0)
Supplement: Supplementary file 1 — Supplementary table [file 41598_2018_20406_MOESM1_ESM.docx]

**Age-related changes in function and gene expression of the male and female mouse bladder**

Jun Kamei, Hiroki Ito, Naoki Aizawa, Harumi Hotta, Toshio Kojima, Yasunori Fujita, Masafumi Ito^5^, Yukio Homma, and Yasuhiko Igawa

Supplementary table. Primer list for real-time RT-PCR analysis

| Gene | Sequence |
| --- | --- |
| M1 receptor | 5′-AGGGATGCGGCAAACTGGTA-3′ |
|  | 5′-AGGTACAGGGTAAGACCTGGGTGA-3′ |
| M2 receptor | 5′-CCAAACCGGTCCAACCTGAG-3′ |
|  | 5′-TGTTCAGTAGTCAAGTGGCCAAAGA-3′ |
| M3 receptor | 5′-TGCTGAGCAGATGGACCAAGA-3′ |
|  | 5′-CGGCAGCTTGAGTACAATGGAA-3′ |
| Beta 1 receptor | 5′-CCGAAAGCAGGTGAATGCAA-3′ |
|  | 5′-TGAAGGTGCATTAAACACACGAAAG-3′ |
| Beta 2 receptor | 5′-TAGCAACGGCAGAACGGACTAC-3′ |
|  | 5′-GGGAGTCAACGCTAAGGCTAGG-3′ |
| Beta 3 receptor | 5′-AAACAAGCGGGTGTCTCCAG-3′ |
|  | 5′-GGAACAGCGTCCAATAGTCACAA-3′ |
| P2X1 receptor | 5′-TCTGGGATTGGCATCTTTGGA-3′ |
|  | 5′-CTCTTAGGCAGGATGTGGAGCAATA-3′ |
|  |  |
| Gapdh | 5′-TGTGTCCGTCGTGGATCTGA-3′ |
|  | 5′-TTGCTGTTGAAGTCGCAGGAG-3′ |
